# Conservation of alternative polyadenylation patterns in mammalian genes

**DOI:** 10.1186/1471-2164-7-189

**Published:** 2006-07-26

**Authors:** Takeshi Ara, Fabrice Lopez, William Ritchie, Philippe Benech, Daniel Gautheret

**Affiliations:** 1 INSERM ERM 206, Université de la Méditerranée, Luminy Case 906, 13288 Marseille, Cedex 09, France; 2 Institut de Génétique et Microbiologie, Université Paris-Sud – CNRS UMR 8621, Bât 400, 91405 Orsay Cedex, France

## Abstract

**Background:**

Alternative polyadenylation is a widespread mechanism contributing to transcript diversity in eukaryotes. Over half of mammalian genes are alternatively polyadenylated. Our understanding of poly(A) site evolution is limited by the lack of a reliable identification of conserved, equivalent poly(A) sites among species. We introduce here a working definition of conserved poly(A) sites as sites that are both (i) properly aligned in human and mouse orthologous 3' untranslated regions (UTRs) and (ii) supported by EST or cDNA data in both species.

**Results:**

We identified about 4800 such conserved poly(A) sites covering one third of the orthologous gene set studied. Characteristics of conserved poly(A) sites such as processing efficiency and tissue-specificity were analyzed. Conserved sites show a higher processing efficiency but no difference in tissular distribution when compared to non-conserved sites. In general, alternative poly(A) sites are species-specific and involve minor, non-conserved sites that are unlikely to play essential roles. However, there are about 500 genes with conserved tandem poly(A) sites. A significant fraction of these conserved tandems display a conserved arrangement of major/minor sites in their 3' UTR, suggesting that these alternative 3' ends may be under selection.

**Conclusion:**

This analysis allows us to identify potential functional alternative poly(A) sites and provides clues on the selective mechanisms at play in the appearance of multiple poly(A) sites and their maintenance in the 3' UTRs of genes.

## Background

Alternative polyadenylation site selection is an important source of transcript diversity in higher eukaryotes. The resulting 3' untranslated region (UTR) variants may differ by their cellular localization, stability or translational efficiency, thus contributing to tissue-specific or developmental stage-specific regulation of gene function [1]. For at least 50% of genes in mammalian genomes, several polyadenylation sites are present and mRNAs with different 3'UTR regions can be produced from a single gene [2-4]. Alternative poly(A) sites are commonly classified into tandem poly(A) sites that locate in the same 3'-exon, and sites located in different exons (including composite exon) formed by alternative splicing [1,4,5]. Alternative 3' ends involving different 3' exons may impact the coding sequence and therefore have obvious functional consequences. However, the actual functional impact of tandem poly(A) sites, producing 3' ends that differ solely by the length of the 3' UTR, is still largely unknown.

Analysis of tissular biases in poly(A) site usage has suggested a frequent tissue-specific regulation of 3' variants in human [6-8]. Recent studies have re-examined alternative polyadenylation in the light of comparative genomics [3-5,7-9]. Features such as the presence of multiple cleavage sites, distribution of poly(A) signal variants and nucleotide composition of flanking regions were reported to be similar in human and mouse [4]. In addition, the numbers and organization of polyadenylation sites in human and mouse orthologs showed significant correlations, suggesting that some alternative polyadenylation patterns are evolutionarily conserved. These studies, however, did not directly address the conservation and functional significance of individual poly(A) sites. Here, we further exploit the tools of comparative genomics to identify and characterize functional alternative polyadenylation sites in the human and mouse genome.

In order to study the evolutionary conservation of poly(A) sites, we need to reliably identify homologs of each alternative poly(A) site in a given gene. We introduce here a method to perform this assignment using both multiple alignments of 3'UTR regions and EST mapping of polyadenylation sites. The functional analysis of conserved and non-conserved poly(A) sites was then carried out based on EST counts and cDNA/EST library information. This resulted in the identification of about 4800 poly(A) sites conserved between human and mouse genes. Comparing the processing efficiency, tissue-specificity and spatial location of conserved and non-conserved poly(A) sites, we identified the characteristic features of conserved sites and estimated the ratio of alternative poly(A) sites under selective pressure. This analysis was complemented by a listing of conserved poly(A) sites with possible tissue-specific usage.

## Results

### Identifying conserved and non-conserved poly(A) sites

We performed a complete mapping of all 3' ESTs and full-length cDNAs onto the human and mouse genomes. After clustering EST and cDNA hits, potential poly(A) sites were identified based on several quality criteria including the presence of at least two ESTs/cDNA ending at site, reduced dangling ends in Blast matches, lack of potential internal priming tract in downstream genomic region and presence of a canonical or variant poly(A) signal near 3' end. We identified a total of 66,647 and 52,270 candidate poly(A) sites in the human and mouse genome, respectively which were then mapped to flanking Ensembl-annotated genes.

Alternative poly(A) site may be found in tandem in the same 3' exon, or in different 3' exons when associated to alternative splicing. We want to avoid the latter case, as poly(A) site usage may be dictated first by alternative splicing, which is itself conserved to some extent for specific genes in animal genomes [10,11]. To avoid interference from alternative splicing, we only considered poly(A) sites located in the 3'-most exon, and following the 3'-most stop codon in case of alternative splice forms. This retained 27,654 poly(A) sites for 14,574 human genes and 25,987 poly(A) sites for 15,199 mouse genes. The resulting estimate of 1.8 poly(A) sites/gene is comparable to previous ones [4,5] with a slight increase for mouse imputable to an expanded mouse EST database.

**Figure 1:**
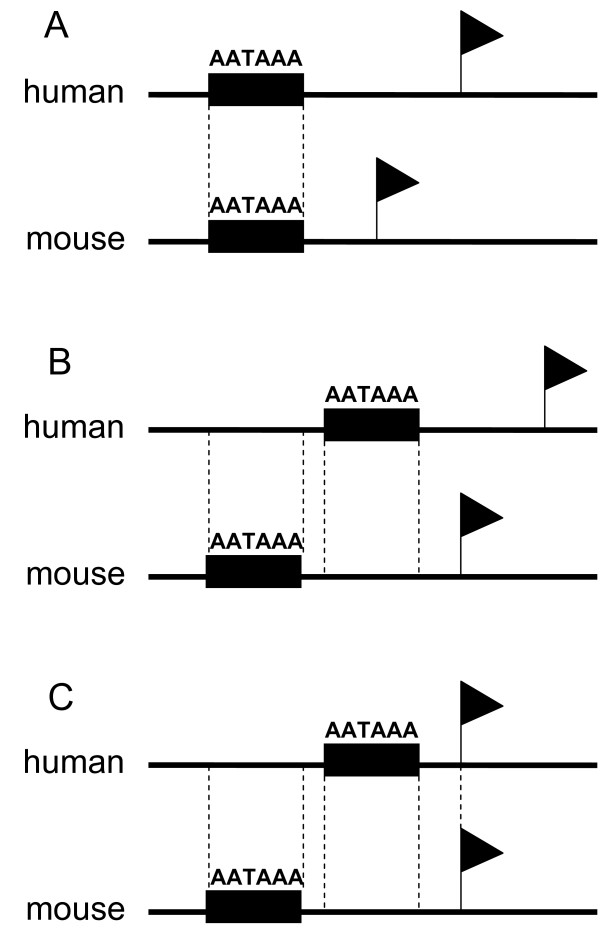
Definition of conserved and non-conserved polyadenylation sites. Cleavage sites are shown by flags and polyadenylation signals are shown by black squares. (A) sites are within 30 bp of each other and aligned associated signals: conserved; (B) sites are within 30 bp of each other, however, their associated signals are not aligned: non conserved; (C) although the sites themselves are aligned, their signals are not: non conserved.

**Figure 2:**
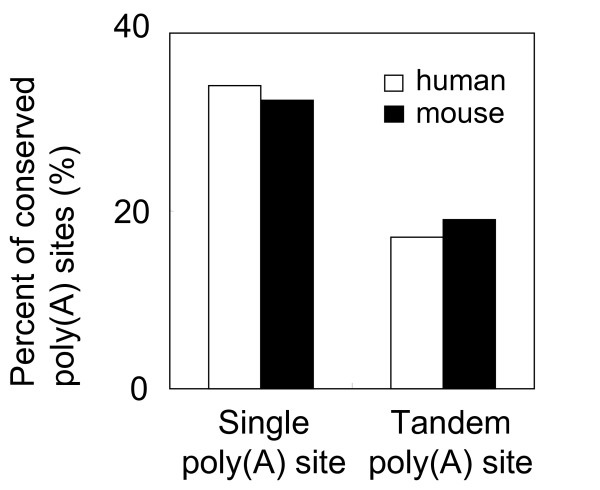
Proportion of conserved sites among single sites and among tandem sites, in human and mouse.

To compare poly(A) sites located on ortholog gene pairs in human and mouse, we aligned 3'UTR regions of all 14,481 ortholog pairs and we defined as "conserved" those human and mouse poly(A) sites displaying aligned poly(A) signals and EST/cDNA support in both species (Figure 1A). Poly(A) sites with EST/cDNA support whose poly(A) signals were not aligned were considered as non-conserved (Figure 1B), even when the cleavage site itself was properly aligned (Figure 1C). Multiple aggregated cleavage sites occurring after a single poly(A) signal (only ~1% of total poly(A) sites in our procedure) were discarded. We obtained a total of 4,807 conserved poly(A) sites and 33,458 non-conserved poly(A) sites. The conserved/non-conserved ratio is about 0.3 in either species. Among conserved poly(A) sites, 3711 were single sites and 1096 were multiple sites from 503 orthologous gene pairs. By our definition, 20% of human genes have a conserved poly(A) site and 2.5% of human genes have multiple conserved poly(A) sites. Figure 2 shows that conservation is higher in single poly(A) sites (33%) than in tandem poly(A) site (18%). This suggests that alternative poly(A) sites are evolutionally less conserved than single poly(A) sites. The complete list of human/mouse conserved poly(A) sites is presented in Additional file 1.

### Number of sites and position in UTR

**Figure 3:**
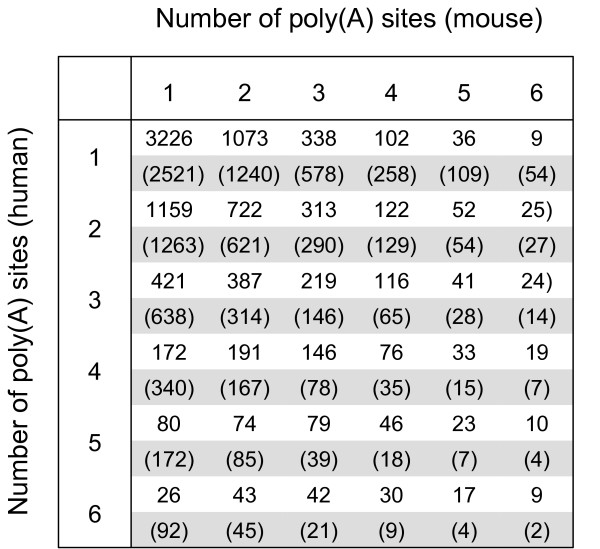
Distribution of ortholog gene pairs against polyadenylation site numbers in human and mouse genes. Table cells provide numbers of orthologous gene pairs in function of the number of polyadenylation sites in human genes (rows) and in mouse genes (columns). Expected values, based on the null hypothesis that there is no correlation, are shown in parentheses. Correlation *P *value: 6.2 × 10^-260 ^(χ^2 ^test).

Tian *et al*. [4] have reported that poly(A) site configurations (single sites, tandem sites or sites located in different exons) tend to be conserved between human and mouse. The conservation of the number of tandem poly(A) sites in homologous gene, however, was not assessed. Figure 3 shows the distribution of poly(A) site numbers in human and mouse orthologous pairs, for genes having one or more sites, conserved or non-conserved. Numbers of poly(A) sites are significantly correlated in orthologs (*P *= 6.2 × 10^-260^, χ^2 ^test). In other words, a human gene with multiple poly(A) sites tends to have multiple poly(A) sites in mouse too, suggesting that a selective mechanism acts on the number of alternative polyadenylation sites.

**Figure 4:**
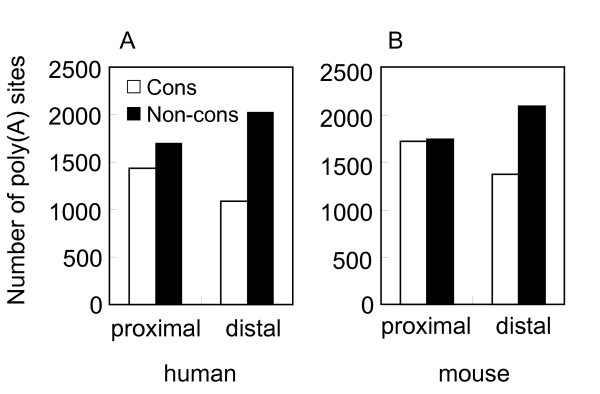
Distribution of polyadenylation sites in proximal or distal parts of 3'UTRs. Numbers of conserved sites ("Cons", white) and non-conserved sites ("Non-cons", black) located in proximal or distal parts of 3'UTR regions are shown for human (A) and mouse (B).

Do conserved tandem poly(A) sites show any positional preference in the 3'UTR region? We examined all tandem poly(A) sites, and classified them as "proximal" or "distal" according to their position relative to the stop codon. For genes with an odd number of sites, the site located in the central position was excluded (10% of sites overall). Figure 4 shows relative locations of conserved and non-conserved tandem poly(A) sites. In both human and mouse, conserved sites tend to occur more often in the proximal part of the UTR, while non-conserved sites tend to occur more often in the distal part.

### Processing efficiency

**Figure 5:**
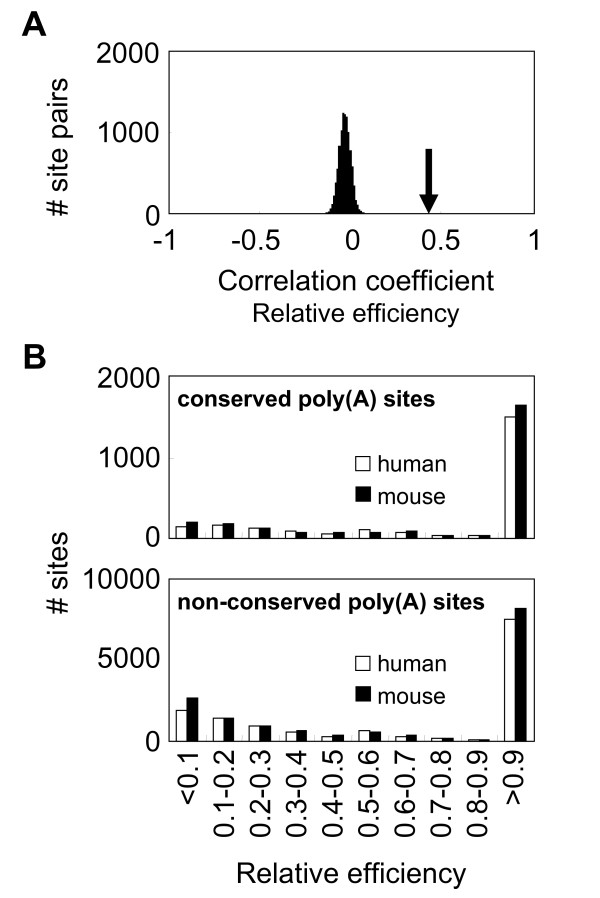
Relative efficiency of conserved and non-conserved polyadenylation sites. (A) Distribution of 10,000 control correlation coefficients each computed from 1000 random pairs of non-conserved sites from orthologous human and mouse genes. Arrow indicates the correlation coefficient observed for pairs of conserved sites (*r *= 0.45). (B) Distribution of relative efficiency of conserved and non-conserved polyadenylation sites. Number of sites in human (white) and mouse (black) is plotted against relative efficiency.

We estimated the relative processing efficiency (RE) of poly(A) sites based on EST counts, normalized in such a way that the highest EST count of all poly(A) sites from the same gene had a value of one. This eliminates biases resulting from different EST coverage in different genes and between human and mouse (see Methods). Figure 5A compares the human/mouse correlation coefficients of RE in conserved poly(A) sites (*r *= 0.45, arrow) and in 10,000 sets of randomly selected non-conserved sites from orthologous genes (histogram). The relative efficiency of conserved poly(A) site is correlated between human and mouse, while that of non-conserved sites from orthologous genes is not.

**Figure 6:**
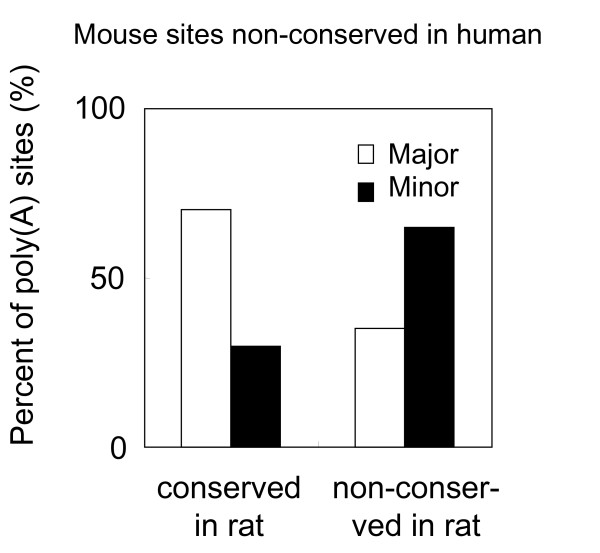
Rat conservation of mouse non-conserved sites. Left: ratio of mouse polyadenylation sites that are non-conserved in the human genome and are conserved in rat. Right: ratio of mouse polyadenylation sites that are non-conserved in the human genome and are non-conserved in rat. Ratios are given for minor (RE<0.5) and major (RE>0.5) sites.

Figure 5B shows the distribution of relative efficiencies in conserved and non-conserved poly(A) sites. If one considers as "major" any site with RE above 0.5, then conserved sites are more often of the major type (85%) than non-conserved sites (75%). This observation raises a question about the nature of conserved sites. Is the higher processing efficiency of conserved sites associated to some selective constraint, or to the presence of non-functional and/or misprediction among non-conserved sites? To answer this question, we subdivided non-conserved mouse sites according to their conservation or lack thereof in rat orthologous genes (Figure 6). Poly(A) sites conserved between mouse and rat behave in the same fashion as poly(A) sites conserved between mouse and human, *i.e*. with a predominance of "major" sites. This suggests that the higher efficiency of conserved human/mouse sites is due firstly to their status of biologically functional sites, rather than to a property of ancient conserved sites.

### Tissue specificity

**Figure 7:**
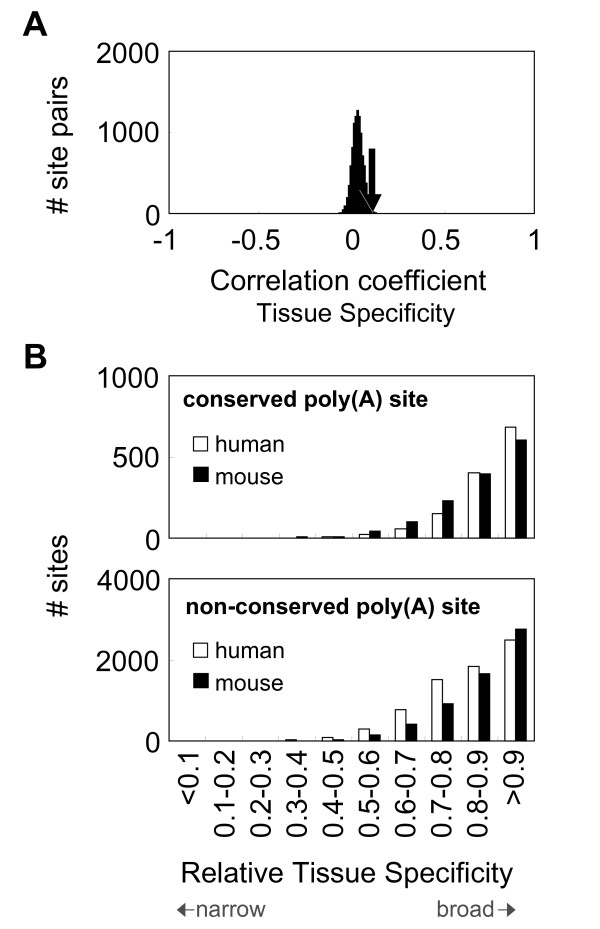
Relative tissue-specificity of conserved and non-conserved polyadenylation sites. (A) Distribution of 10,000 control correlation coefficients each computed from 1000 random pairs of non-conserved sites from orthologous human and mouse genes. Arrow indicates the correlation coefficient observed for pairs of conserved sites (*r *= 0.10). (B) Distribution of relative tissue-specificities in conserved and non-conserved polyadenylation sites. Number of sites in human (white) and mouse (black) is plotted against relative tissue specificity.

We then analyzed the tissue specificity of poly(A) sites based on the eVOC expression ontology system [12]. Version 2.6 of eVOC maps each of the 9,478 human EST libraries to a formalized tissue description. As a mouse version of eVOC was not available at the time of the study, we further mapped 556 mouse EST libraries using the same formal description system (See Materials and Methods). We obtained for each poly(A) site the number of different tissues in which the site is observed, among the 12 possible top-level eVOC tissue categories. Since tissue counts are highly dependent on EST coverage, we normalized tissue counts versus an expected number of tissues obtained from a random EST sample of same size. Our measure of tissue specificity is the log ratio of observed *vs*. expected number of tissues. About 10% of tandem poly(A) sites have a tissue specificity below -0.5 (high specificity) and <3% above 0.5 (low specificity). Non-conserved poly(A) sites showed no correlation in tissue specificity (Fig 7A, histogram), while conserved sites had weakly correlated tissue specificities (*r *= 0.10, Fig 7A, arrow). This observed difference is not significant based on a T-test performed after Fisher's z-transformation of the *r *value (P < 0.5).

To circumvent possible gene-level expression biases, we measured a "relative tissue specificity" (RTS), by assigning a value of 1 to the poly(A) site with the broadest tissue distribution in a gene. Each gene thus has at least one site with RTS = 1. The distribution of other sites is shown in Figure 7B. Interestingly, very few sites have a RTS below 0.5. The fact that most sites have a RTS close to 1, that is close to the broadest possible tissue distribution for this gene, means variations in tissue specificity between successive poly(A) sites in a gene are generally limited. We used the median RTS to distinguish "broad" from "narrow" sites (we preferred these terms over "constitutive" and "specific" since these would suggest an absolute usage level, while here we only measure relative usage). Sites with an RTS above median (0.90 for human, 0.88 for mouse) are said to display a "broad" tissue distribution while other sites are said to display a "narrow" tissue distribution. Based on this definition, broad and narrow tissue distributions are equally frequent among conserved and non-conserved sites (Figure 7B). Broad and narrow tissue distributions are also equally distributed among major and minor sites (Additional file 2).

### Spatial preferences *vs*. efficiency or specificity

**Figure 8:**
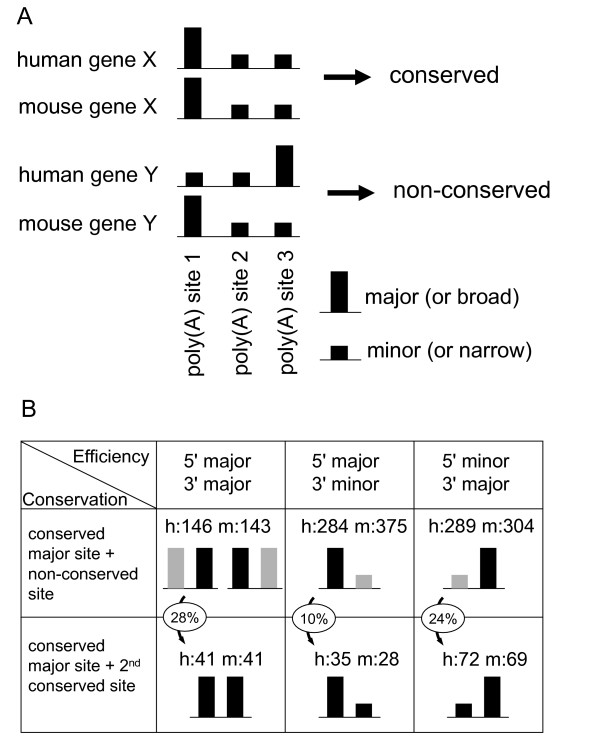
Spatial efficiency/specificity patterns and conservation. (A) Definition of spatial efficiency/specificity patterns. Ortholog gene pairs with identical numbers of conserved polyadenylation sites are considered. A conserved pattern is defined as a series of sites between two orthologous genes, where each site bares the same properties (major/minor or broad/narrow) as its orthologous counterpart (e.g. gene X). All other patterns are defined as non-conserved (e.g. gene Y). (B) Relationship between tandem poly(A) site spatial patterns and conservation. The first row shows different patterns in which one major site is conserved. The second row shows different patterns in which two sites are conserved. Numbers of human genes ("h") and mouse genes ("m") displaying each pattern are shown. Circled numbers indicate ratio of patterns in second row over patterns in first row.

We examined the relationship between the spatial organization of tandem poly(A) sites and site efficiency or specificity. Poly(A) sites were classified as major/minor or narrow/broad as described above, and their spatial organization was observed in genes containing two or more tandem conserved sites. A "conserved usage pattern" was recorded when successive tandem sites had the same efficiency and/or specificity pattern in human and mouse orthologs (Figure 8A). We observed that 53% of genes with tandem conserved poly(A) sites had a conserved efficiency pattern (227 gene pairs), significantly higher than expected by chance (146 gene pairs, binomial distribution *P *= 9.7 × 10^-28^). Comparing expected and observed values, 81 genes would have a poly(A) site efficiency pattern under selection. This tendency is not observed for tissue specificity: 133 gene pairs have a conserved tissue specificity pattern *vs*. 141 expected by chance.

In Figure 8B, we focus on genes containing at least one conserved major site, as a surrogate for sites existing prior to human-mouse divergence. We then observe how such sites are associated to flanking conserved (black) or non-conserved (gray) sites, using flanking non-conserved sites as surrogates for emerging sites. Interestingly, while emerging minor sites are as frequent on the 5' or 3' side of existing major sites (top row), pairs of conserved sites (bottom row) are twice more often 5'minor/3'-major than 5'-major/3'-minor. This suggests that selection of alternative poly(A) sites favors the pattern 5'-minor/3'-major over the pattern 5'-major/3'-minor.

### Differentially processed Poly(A) sites

We define here as differentially processed those alternative poly(A) sites with a significantly biased usage in any tissue class, as compared with other poly(A) sites from the same gene. Differential usage was measured using a Fisher test as previously reported [6]. Using a Bonferroni correction for multiple testing, five conserved and 84 non-conserved sites are differentially processed in human (54 and 369, respectively, in mouse).

**Figure 9:**
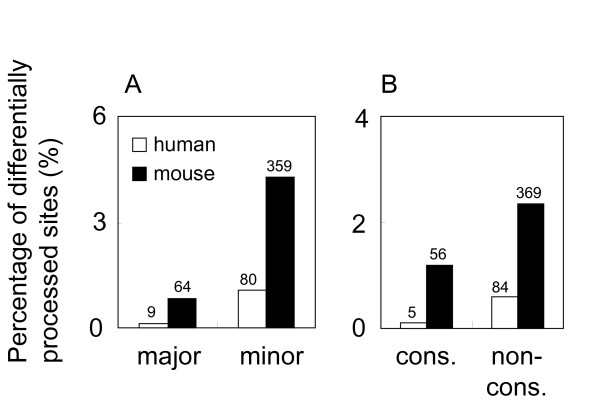
Distribution of differentially-processed polyadenylation sites. (A) Ratio of differentially-processed sites (*P *< 0.05, Fisher's exact test with Bonferroni correction) against total sites, for human (white) and mouse (black) genes, shown for major and minor sites. (B) Ratio of differentially-processed sites (*P *< 0.05, Fisher's exact test with Bonferroni correction) against total sites, shown for conserved and non-conserved sites. Value above each bar show absolute numbers of differentially-processed sites.

We examined the relationship between differential processing and site efficiency or tissue specificity. Consistently with a recent study of tissue-specific polyadenylation [8], minor sites are more often differentially processed than major sites (Figure 9A). Although this study did not identify tissue biases in major sites, we do observe a few occurrences (9 in human, 64 in mouse) of major sites with differential processing. Differential processing is also much more frequent in non-conserved than in conserved sites (Figure 9B).

Expectedly, there is a high correlation between differential usage and the "narrow" or "broad" status of a site. Differentially processed sites are about three times more often of the narrow type than of the broad type (data not shown). Although counterintuitive, some poly(A) sites can be at the same time differentially processed and of broader usage, because our specificity measure is relative and always classifies as broad the site with the broadest tissue usage, even when usage is restricted to a single tissue.

**Figure 10:**
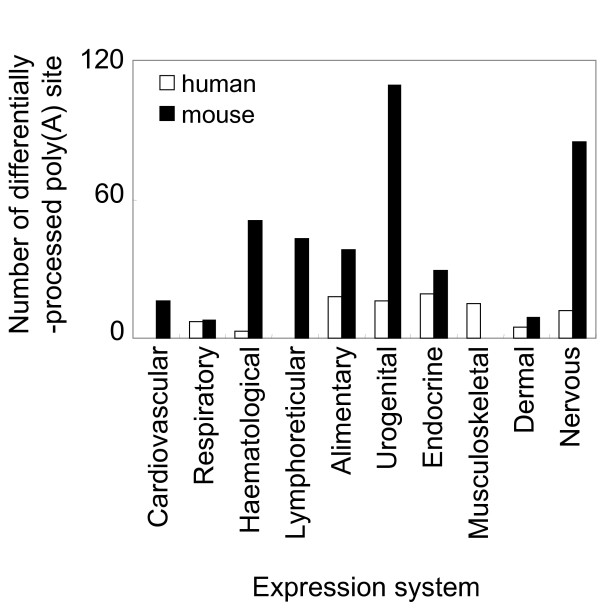
Tissue-distribution of differentially-processed, polyadenylation sites, in human (white) and mouse (black).

A list of differentially processed, conserved poly(A) sites is presented in Additional file 3. Differentially processed sites are observed in all tissue classes (Figure 10). The apparent overrepresentation of urogenital and nervous systems is not significant when EST library coverage is taken into account. EST coverage is not sufficient either to provide interspecies confirmation of tissue biases. No conserved site is found differentially processed in both human and mouse after Bonferroni correction.

### Conserved sequence motifs around conserved sites

As our criteria for poly(A) site conservation imply a correct alignment of poly(A) signals, we suspected that conserved sequences around poly(A) signals could also contribute to poly(A) site conservation. This region is known to contain elements such as the USE (upstream sequence element) and DSE (downstream sequence element), two U-rich elements involved in the control of poly(A) site efficiency [13-15], as well as a number of potential regulatory motifs of unknown function [7]. A possible explanation for proper signal alignment and increased cleavage efficiency at conserved poly(A) sites could be related to the occurrence of such control elements in both human and mouse orthologs. Although downstream regions appear slightly more U-rich in conserved sites than in non-conserved sites (see Additional file 4), indicative of stronger DSE elements [15], we could not find overrepresented sequence motifs occurring in more than a few conserved sites. Therefore, there is no widespread cis-regulatory element that would explain poly(A) site conservation.

## Discussion

We introduced here a definition of conserved poly(A) sites as sites supported by 3' ESTs or full length cDNAs in ortholog gene pairs and located downstream of a properly aligned AAUAAA or variant signal in the pairwise 3' UTR alignment. Applying this rule to human and mouse orthologs, we observed 4,807 conserved poly(A) sites, *i*.e. about 22% of the human sites tested. Only a third of the human/mouse orthologous gene pairs contains one or more conserved sites by this definition.

Gene Ontology (GO) term analysis suggests links between alternative polyadenylation and specific functions. As previously reported [4], genes with tandem poly(A) sites are enriched in terms "intracellular" (cellular component; GO:0005622) and "protein transport" (biological process; GO:0015031). Now, if all tandem sites are used as a reference, genes with *conserved *tandem sites are further enriched for terms "nucleus" (cellular component; GO:0005634, number of gene n = 129, *P *= 5.5 × 10^-5 ^for human and n = 125, *P *= 1.0 × 10^-7 ^for mouse) and "ubiquitin cycle" (biological process; GO:0006512, n = 27, *P *= 6.2 × 10^-6 ^for human and n = 23, *P *= 8.4 × 10^-5 ^for mouse). The nucleus encompasses evolutionally conserved DNA and RNA processing machineries. Alternative polyadenylation may be more conserved in genes within such cellular systems. The ubiquitin cycle is also well conserved among eukaryotic genomes and involves genes containing highly conserved 3' UTR elements in vertebrates [16]. This is consistent with posttranscriptional regulations involving this region and hence with a selective pressure for conserved tandem poly(A) sites.

Among genes with tandem poly(A) sites (70% of our mapped gene set), the most frequent patterns involve either only non-conserved sites (~3000 genes) or a single conserved site flanked by non-conserved sites (~2000 genes). There are only about 500 genes with two or more conserved poly(A) sites. When comparing the efficiency and specificity of poly(A) sites in a tandem configuration, a general picture emerges where conserved sites generally show a higher efficiency and fewer instances of differential processing than non-conserved sites. The majority of minor or tissue-specific sites are non-conserved, suggesting that alternative polyadenylation is most frequently a species-specific event. This is reminiscent of what was observed for alternative splicing. Modrek *et al*. [10] reported that, for skipped exons, major forms are more often conserved than minor forms, thus suggesting that alternative splicing is more often species-specific as well.

We found that processing efficiency was significantly correlated between human and mouse at conserved sites. Again, this pattern is reminiscent of that observed for alternative splicing. Looking at conserved alternative splicing events, Kan *et al*. observed strong correlations of human/mouse expression levels that were suggestive of functional alternative splicing events [11].

We observed that the spatial organization of major/minor poly(A) sites in a gene is conserved more often than expected by chance. This suggests that, for some genes, specific usage patterns of alternative poly(A) sites were established prior to the human/mouse divergence and were maintained by selection. We estimate this should concern no more than one hundred genes.

The large number of non-conserved poly(A) sites, especially among tandem sites, suggests that gain/loss of alternative poly(A) sites is a frequent event in mammalian genomes. New poly(A) signals may arise from duplications, insertion events or point mutations. The latter is probably a more parsimonious hypothesis when considering the AU-rich nature of non-coding human sequences and the presence in UTRs of AU-rich elements such as the AREs, resembling poly(A) signals. However, new signal arising from point mutation are most likely deprived from enhancing elements and hence should produce transcript isoforms in very small quantities, especially if located downstream of a strong site. On the other hand, poly(A) sites resulting from duplication or insertion of a functional signal and its associated enhancing elements maybe readily functional and able to compete effectively with alternative sites.

Are new alternative sites selectively neutral and what is their fate? Novel 3' variants can be non-neutral for instance when containing regulatory motifs such as miRNA targets or destabilization elements, or when affecting translation efficiency through the sheer effect of 3' UTR size [17]. Our observation that tandem poly(A) sites are generally less conserved than unique sites suggests that most novel sites are quickly lost and therefore are either neutral or deleterious. Interestingly, spatial patterns of the type 5'-major/3'-minor are underrepresented in conserved tandem sites (Figure 8B). This is consistent with a model where novel poly(A) sites arising 3' to existing sites tend to be lost more quickly, unless stronger than existing 5' sites. Through the accidental occurrence and loss of novel poly(A) sites in the 3' UTR, natural selection would thus tend towards a topology involving a minor short isoform and a major long isoform, which is indeed the most frequent topology observed for polyadenylation isoforms [2].

## Conclusion

We used comparative genomics to identify and characterize functional polyadenylation sites in the human and mouse genomes. A genome-wide computational analysis of alternative polyadenylation sites allowed us to identify about 4800 conserved poly(A) sites. Conserved sites display a higher processing efficiency than non-conserved sites, but display no difference in tissue distribution. We focused on tandems of conserved sites and sought biases in site usage and position in UTR. The 5'/3' order of major and minor sites in conserved tandems is more conserved than expected by chance, suggesting that selective pressure acts on poly(A) site usage and therefore that resulting alternative transcripts may have functional significance. Some unanticipated patterns deserve further scrutiny, such as major sites with a predicted differential usage, or conserved sites that yet are of the minor or tissue-specific type. Transcripts displaying such unexpected poly(A) site usage patterns could be prioritized for experimental validation.

## Methods

### Poly(A) site prediction

EST sequences were obtained from dbEST v. 01/06/05 (6,009,051 human, 4,314,509 mouse and 623,741 rat ESTs). Full-length cDNA sequences were obtained from H-Inv 1.8 (41,118 sequences) [18] and FANTOM 2.01 (60,770 sequences) [19]. ESTs annotated as 3' were extracted (2,029,534 human, 1,864,874 mouse and 293,597 rat ESTs) and trailing poly(A) or poly(T) sequences of 5 nt or more were removed, with one mismatch (non-A or non-T) allowed for polyA/T tails of 10 or more. Both 3' EST and cDNA sequences were aligned to the repeat-masked human genome v27.35a.1, mouse genome v27.33c.1 and rat genome v27.3e.1 using the Megablast program [20]. We did not use a specific exon junction mapping software, since we were only interested in the terminal part of the 3' exon. All hits presenting at least 95% identity with the genomic sequence were retained (hit size >28 nt at default E-value). Partial hits flanking a repeat masked region of the genome were then realigned to the locally unmasked region. Hits with 95% identity after this step were retained. Clusters were formed with ESTs having either their 5' or 3' extremities falling within a 10 nt distance. ESTs were not oriented at this stage. Each cluster was analyzed using a sliding window to locate the most likely cleavage site, defined as the position where the window contains the most EST/cDNA ends. The following filters where then applied:

(i) Dangling ends: discard hits with more than 5 unmatched nt at cleavage site

(ii) Internal priming: discard cleavage sites flanked by A-rich region (at least 9 As out of 10 nt) in the 50 nt downstream genomic sequence

(iii) Poly(A) signal: retain only cleavage sites where 30 nt upstream genomic sequence contains one of the 11 variant poly(A) signal identified in our previous study [2]: AATAAA, ATTAAA, AGTAAA, TATAAA, CATAAA, GATAAA, AATATA, AATACA, AATAGA, AATGAA, ACTAAA

Only those cleavage sites passing the filters and supported by at least two ESTs/cDNAs were retained as predicted poly(A) sites. From the starting EST/cDNA datasets we finally retained 718,927 ESTs and 19,626 full length cDNAs to identify 66,647 different poly(A) sites in human, 739,259 ESTs and 23,069 full length cDNAs to identify 52,232 different poly(A) sites in mouse and 119,946 ESTs to identify 27,494 different poly(A) sites in rat.

### Assignment of tandem Poly(A) signal sites

Poly(A) sites were assigned to transcript sequences taken from Ensembl 27.35a.1 [21]. If the poly(A) site lied within one or more annotated transcripts, downstream of the end of translation, the site was affected to each of these transcripts. If the poly(A) site lied upstream of the end of translation, then it was considered as "in CDS" and is not used for analysis. If the poly(A) site did not map to any annotated transcript, it was affected to the nearest 5' transcript. Poly(A) signals were assigned to their respective poly(A) sites by taking the signal that was closest to the 5'-most poly(A) site in each cluster. Only poly(A) sites mapping to the 3'-most exon of an Ensembl gene or its genomic downstream region up to 10 kb were considered further.

### Assignment of conserved Poly(A) sites

Ortholog human/mouse (or mouse/rat) gene pairs were obtained from EnsMart [22]. All genes with paralogs were omitted from the analysis. 3'UTR regions assigned in Ensembl including up to 10 kb downstream genomic sequence of all transcripts were aligned by ClustalW with default parameter. Predicted Poly(A) sites were then defined as conserved if they were within a distance of 30 bp of a properly aligned poly(A) signal and had EST-support in both human and mouse. In the case where multiple poly(A) signals were associated to a single cleavage site, the signal closest to the cleavage site was used for the analysis. Multiple cleavage sites that were associated to the same poly(A) signal were omitted.

### Mouse eVOC ontology mapping

Anatomical terms in mouse cDNA libraries were mapped to anatomical systems from the eVOC ontology ver 2.6 [12]. Terms that matched exactly to human eVOC terms were kept and all mixed terms (e.g. lung and colon) were manually checked to see if each component could match to a human eVOC term. Any terms that could not be directly mapped were classified as "unclassifiable". Finally all libraries were assigned to 12 anatomical systems: anatomical site, cardiovascular, respiratory, hematological, lymphoreticular, alimentary, urogenital, endocrine, musculoskeletal, dermal, nervous and unclassifiable.

### Correlation analysis

χ^2 ^test, t-test, calculation of correlation coefficient and Fisher's z-transformation were performed using Microsoft Excel 2002. Distributions of observed and random distributed values were calculated using dedicated Perl scripts.

### Efficiency and tissue specificity

All EST counts were performed after discarding EST libraries annotated as normalized in the dbEST database (3% of overall human and mouse libraries). The relative efficiency *R *of a poly(A) site was calculated as a ratio of number of ESTs,

Ri=nX,inX,max⁡
 MathType@MTEF@5@5@+=feaafiart1ev1aaatCvAUfKttLearuWrP9MDH5MBPbIqV92AaeXatLxBI9gBaebbnrfifHhDYfgasaacH8akY=wiFfYdH8Gipec8Eeeu0xXdbba9frFj0=OqFfea0dXdd9vqai=hGuQ8kuc9pgc9s8qqaq=dirpe0xb9q8qiLsFr0=vr0=vr0dc8meaabaqaciaacaGaaeqabaqabeGadaaakeaacqWGsbGudaWgaaWcbaGaemyAaKgabeaakiabg2da9maalaaabaGaemOBa42aaSbaaSqaaiabdIfayjabcYcaSiabdMgaPbqabaaakeaacqWGUbGBdaWgaaWcbaGaemiwaGLaeiilaWIagiyBa0MaeiyyaeMaeiiEaGhabeaaaaaaaa@3D5F@

where *n*_*x*,*i *_is the number of ESTs of poly(A) site *i *within gene *X*, and *n*_*X*,*max *_is maximum number of ESTs of any tandem poly(A) site within gene X.

Sites with a ratio higher than 0.5 were defined as "major" (high efficiency), while other sites were defined as "minor" (low efficiency).

The specificity of a poly(A) site was defined as the number of different expression systems in which this site was utilized, normalized by the number of supporting ESTs as follows. For a poly(A) site supported by *N *ESTs, we calculated an expected number of expression systems as the average number of expression systems obtained in 10,000 random sets of *N *ESTs sampled from the complete set of ESTs mapping any poly(A) site. Specificity S was then calculated as the log-ratio of the number of expression systems:

S=log⁡(TTsim[n])
 MathType@MTEF@5@5@+=feaafiart1ev1aaatCvAUfKttLearuWrP9MDH5MBPbIqV92AaeXatLxBI9gBaebbnrfifHhDYfgasaacH8akY=wiFfYdH8Gipec8Eeeu0xXdbba9frFj0=OqFfea0dXdd9vqai=hGuQ8kuc9pgc9s8qqaq=dirpe0xb9q8qiLsFr0=vr0=vr0dc8meaabaqaciaacaGaaeqabaqabeGadaaakeaacqWGtbWucqGH9aqpcyGGSbaBcqGGVbWBcqGGNbWzdaqadiqaamaalaaabaGaemivaqfabaGaemivaq1aaSbaaSqaaiabdohaZjabdMgaPjabd2gaTjabcUfaBjabd6gaUjabc2faDbqabaaaaaGccaGLOaGaayzkaaaaaa@3F44@

where *T *is observed number of different expression system in each poly(A) site, *T*_*sim [n] *_is the simulated number of different expression systems for *n *ESTs.

For genes with tandem poly(A) sites, a relative tissue specificity *U *was calculated for each site, as the ratio of tissue specificities:

Ui=SX,i−Smin⁡SX,max⁡−Smin⁡
 MathType@MTEF@5@5@+=feaafiart1ev1aaatCvAUfKttLearuWrP9MDH5MBPbIqV92AaeXatLxBI9gBaebbnrfifHhDYfgasaacH8akY=wiFfYdH8Gipec8Eeeu0xXdbba9frFj0=OqFfea0dXdd9vqai=hGuQ8kuc9pgc9s8qqaq=dirpe0xb9q8qiLsFr0=vr0=vr0dc8meaabaqaciaacaGaaeqabaqabeGadaaakeaacqWGvbqvdaWgaaWcbaGaemyAaKgabeaakiabg2da9maalaaabaGaem4uam1aaSbaaSqaaiabdIfayjabcYcaSiabdMgaPbqabaGccqGHsislcqWGtbWudaWgaaWcbaGagiyBa0MaeiyAaKMaeiOBa4gabeaaaOqaaiabdofatnaaBaaaleaacqWGybawcqGGSaalcyGGTbqBcqGGHbqycqGG4baEaeqaaOGaeyOeI0Iaem4uam1aaSbaaSqaaiGbc2gaTjabcMgaPjabc6gaUbqabaaaaaaa@49E1@

where *S*_*X*,*i *_is tissue specificity of each tandem poly(A) site *i *within gene X, *S*_*X*,*max *_is maximum tissue specificity of tandem poly(A) site within gene X, and *S*_*min *_is minimum value of *S *in each species. We adjusted the interval of relative tissue specificity to 0–1; 0 being the most specific site and 1 being the least specific. Median ratio was 0.90 for human, 0.88 for mouse. Sites with a ratio higher than these values were defined as "broad" while others were defined as "narrow".

A usage pattern was defined as the sequence of relative efficiencies or relative tissue specificities for all tandem poly(A) sites in a gene. For each usage pattern, the expected value E of the number of gene pairs that could randomly bare this pattern was calculated from a random combination of human and mouse gene pairs with conserved tandem poly(A) sites as described below. For a number of conserved poly(A) sites *j*, the list of relative usage patterns *L *[j, k] was defined by

*L *[j, k] = {[r_1_, r_2_,...,r_j_]_1_, [r_1_, r_2_,...,r_j_]_2_, ..., [r_1_, r_2_,..., r_j_]_k_}

where r_j _= {major or minor} or {broad or narrow}. For a number *N *of genes and the number of conserved poly(A) sites j, a probability *P *for human and mouse poly(A) sites having pattern *L *[j, k] was defined as:

Phuman=NL[j,k],humanNtotal,Pmouse=NL[j,k],mouseNtotal
 MathType@MTEF@5@5@+=feaafiart1ev1aaatCvAUfKttLearuWrP9MDH5MBPbIqV92AaeXatLxBI9gBaebbnrfifHhDYfgasaacH8akY=wiFfYdH8Gipec8Eeeu0xXdbba9frFj0=OqFfea0dXdd9vqai=hGuQ8kuc9pgc9s8qqaq=dirpe0xb9q8qiLsFr0=vr0=vr0dc8meaabaqaciaacaGaaeqabaqabeGadaaakeaacqWGqbaudaWgaaWcbaGaeeiAaGMaeeyDauNaeeyBa0MaeeyyaeMaeeOBa4gabeaakiabg2da9maalaaabaGaemOta40aaSbaaSqaaiabdYeamjabcUfaBjabdQgaQjabcYcaSiabdUgaRjabc2faDjabcYcaSiabdIgaOjabdwha1jabd2gaTjabdggaHjabd6gaUbqabaaakeaacqWGobGtdaWgaaWcbaGaemiDaqNaem4Ba8MaemiDaqNaemyyaeMaemiBaWgabeaaaaGccqGGSaalcqWGqbaudaWgaaWcbaGaeeyBa0Maee4Ba8MaeeyDauNaee4CamNaeeyzaugabeaakiabg2da9maalaaabaGaemOta40aaSbaaSqaaiabdYeamjabcUfaBjabdQgaQjabcYcaSiabdUgaRjabc2faDjabcYcaSiabd2gaTjabd+gaVjabdwha1jabdohaZjabdwgaLbqabaaakeaacqWGobGtdaWgaaWcbaGaemiDaqNaem4Ba8MaemiDaqNaemyyaeMaemiBaWgabeaaaaaaaa@71A4@

Then the expected value *E *for the maximal value of *k *(k_max _= 2^j^-1) is :

E=∑j=1jmax⁡{∑k=1kmax⁡(Nj∗Phuman∗Pmouse)}
 MathType@MTEF@5@5@+=feaafiart1ev1aaatCvAUfKttLearuWrP9MDH5MBPbIqV92AaeXatLxBI9gBaebbnrfifHhDYfgasaacH8akY=wiFfYdH8Gipec8Eeeu0xXdbba9frFj0=OqFfea0dXdd9vqai=hGuQ8kuc9pgc9s8qqaq=dirpe0xb9q8qiLsFr0=vr0=vr0dc8meaabaqaciaacaGaaeqabaqabeGadaaakeaacqWGfbqrcqGH9aqpdaaeWbqaamaacmqabaWaaabCaeaadaqadiqaaiabd6eaonaaBaaaleaacqWGQbGAaeqaaOGaey4fIOIaemiuaa1aaSbaaSqaaiabdIgaOjabdwha1jabd2gaTjabdggaHjabd6gaUbqabaGccqGHxiIkcqWGqbaudaWgaaWcbaGaemyBa0Maem4Ba8MaemyDauNaem4CamNaemyzaugabeaaaOGaayjkaiaawMcaaaWcbaGaem4AaSMaeyypa0JaeGymaedabaGaem4AaS2aaSbaaWqaaiGbc2gaTjabcggaHjabcIha4bqabaaaniabggHiLdaakiaawUhacaGL9baaaSqaaiabdQgaQjabg2da9iabigdaXaqaaiabdQgaQnaaBaaameaacyGGTbqBcqGGHbqycqGG4baEaeqaaaqdcqGHris5aaaa@5E4C@

Expected frequencies of genes with narrow usage patterns, based on a binomial distribution, were calculated by Microsoft Excel 2002.

### Differential use of poly(A) sites

To identify differentially processed poly(A) sites, Fisher's tests were performed on the distribution of the number of supporting ESTs from each expression system against all other systems for each poly(A) site as previously described [6]. A Bonferroni correction for multiple testing was applied. Poly(A) sites supported only by ESTs from pooled tissue libraries were omitted.

## Authors' contributions

TA conceived the study, performed computational analyzes and drafted the manuscript. FL performed poly(A) site mapping and annotation. WR contributed to data analysis. PB contributed to scientific directions and writing of the manuscript. DG directed the study and co-wrote the manuscript. All authors read and approved the final manuscript.

## Supplementary Material

Additional File 1 List of human/mouse conserved polyadenylation sites (4,807 sites). Click here for file

Additional File 2 Number of tandem poly(A) sites in different expression categories. Click here for file

Additional File 3 List of differentially processed, conserved polyadenylation sites in human (5 sites) and mouse (54 sites). Click here for file

Additional File 4 Nucleotide frequencies in downstream regions of (a) poly(A) sites in VEGA genes, (b) our predicted poly(A) sites, split into (c) conserved and (d) non-conserved sites; and (e) randomly occurring AAUAAA signals in 3' UTRs. Click here for file
