# Supplementary material for: Conservation of alternative polyadenylation patterns in mammalian genes
Source: BMC Genomics. 2006 Jul 26;7:189. doi: 10.1186/1471-2164-7-189 (PMC1550727; doi:10.1186/1471-2164-7-189)
Supplement: Additional File 4 — Nucleotide frequencies in downstream regions of (a) poly(A) sites in VEGA genes, (b) our predicted poly(A) sites, split into (c) conserved and (d) non-conserved sites; and (e) randomly occurring AAUAAA signals in 3' UTRs. [file 1471-2164-7-189-S4.ppt]

## Slide 1
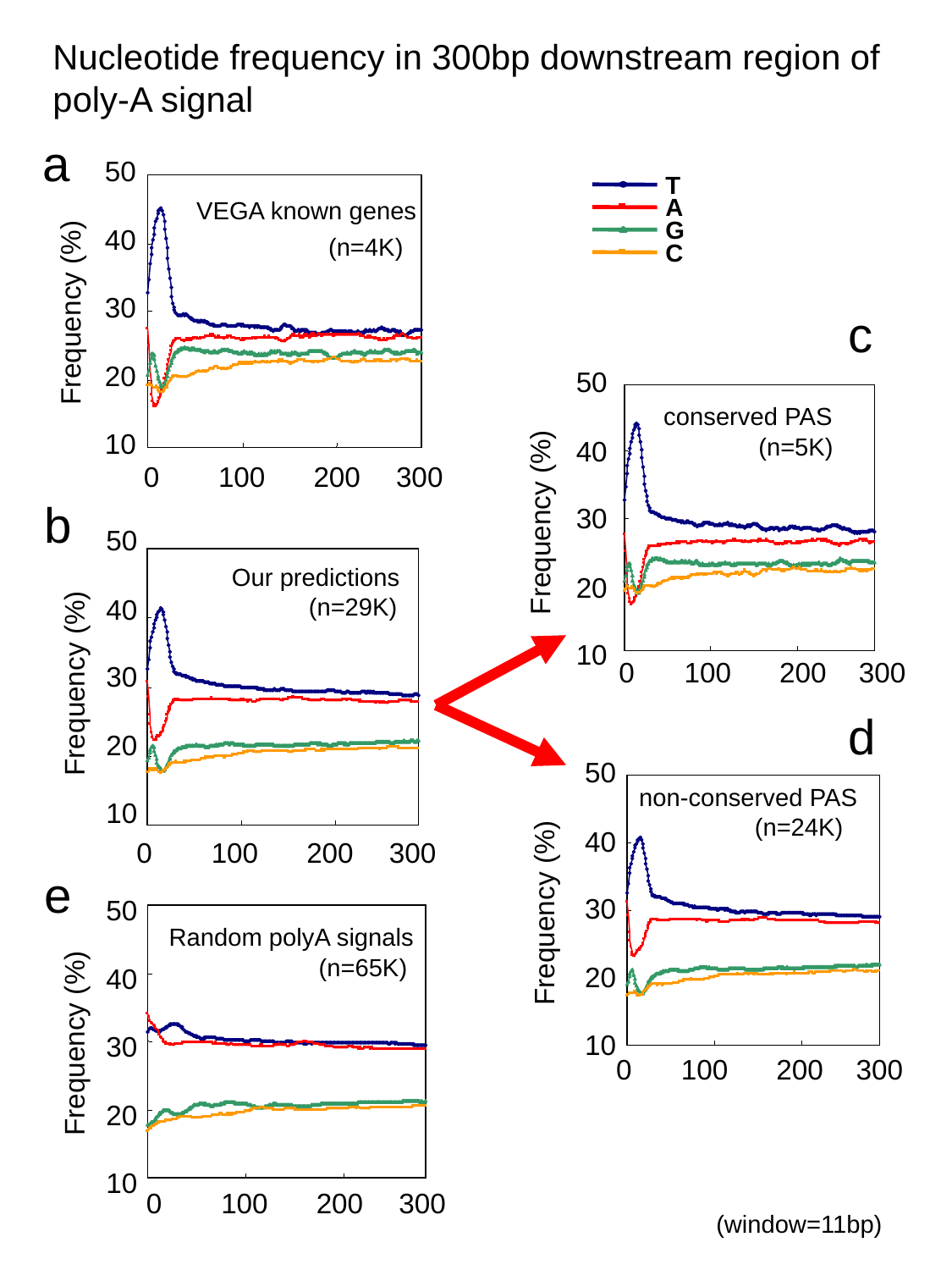

Nucleotide frequency in 300bp downstream region of poly-A signal
a
50
T
A
G
C
VEGA known genes
40
(n=4K)
30
Frequency (%)
c
20
50
conserved PAS
(n=5K)
10
40
0
100
200
300
b
30
Frequency (%)
50
Our predictions
20
(n=29K)
40
10
0
100
200
300
30
Frequency (%)
d
20
50
non-conserved PAS
10
(n=24K)
40
0
100
200
300
e
30
50
Frequency (%)
Random polyA signals
(n=65K)
20
40
Frequency (%)
10
30
0
100
200
300
20
10
0
100
200
300
(window=11bp)
